# Architectural control of mesenchymal stem cell phenotype through nuclear actin

**DOI:** 10.1080/19491034.2022.2029297

**Published:** 2022-02-08

**Authors:** Janet Rubin, Andre J. van Wijnen, Gunes Uzer

**Affiliations:** a Department of Medicine, University of North Carolina, Chapel Hill, NC, USA; b Department of Biochemistry, University of Vermont Medical School, Burlington, Vt, USA; c Department of Mechanical & Biomedical Engineering, Boise State University, Boise, ID, USA

**Keywords:** Mechanoresponse, mechanical-strain, mesenchymal stem cell, cofilin, Arp2/3, formin, epigenetics

## Abstract

There is growing appreciation that architectural components of the nucleus regulate gene accessibility by altering chromatin organization. While nuclear membrane connector proteins link the mechanosensitive actin cytoskeleton to the nucleoskeleton, actin’s contribution to the inner architecture of the nucleus remains enigmatic. Control of actin transport into the nucleus, plus the presence of proteins that control actin structure (the actin tool-box) within the nucleus, suggests that nuclear actin may support biomechanical regulation of gene expression. Cellular actin structure is mechanoresponsive: actin cables generated through forces experienced at the plasma membrane transmit force into the nucleus. We posit that dynamic actin remodeling in response to such biomechanical cues provides a novel level of structural control over the epigenetic landscape. We here propose to bring awareness to the fact that mechanical forces can promote actin transfer into the nucleus and control structural arrangements as illustrated in mesenchymal stem cells, thereby modulating lineage commitment.

## Introduction

Actin in the cytoplasm provides structure to the cell, dynamically remodeling cellular structure to allow cell division, compartmentalization of cellular organelles, scaffolding of signaling components, and cell motility. Actin structure also contributes to the ability of the cell to sense its microenvironment, particularly the local mechanical environment [1–3]. More recently, actin connections to the nuclear membrane and trafficking of mechanically activated proteins into the nucleus in response to mechanical force have brought an appreciation that the nucleus itself responds to mechanical input transmitted from the substrate through the cell body and provides regulatory control of cell function through gene expression.

**Figure 1.**
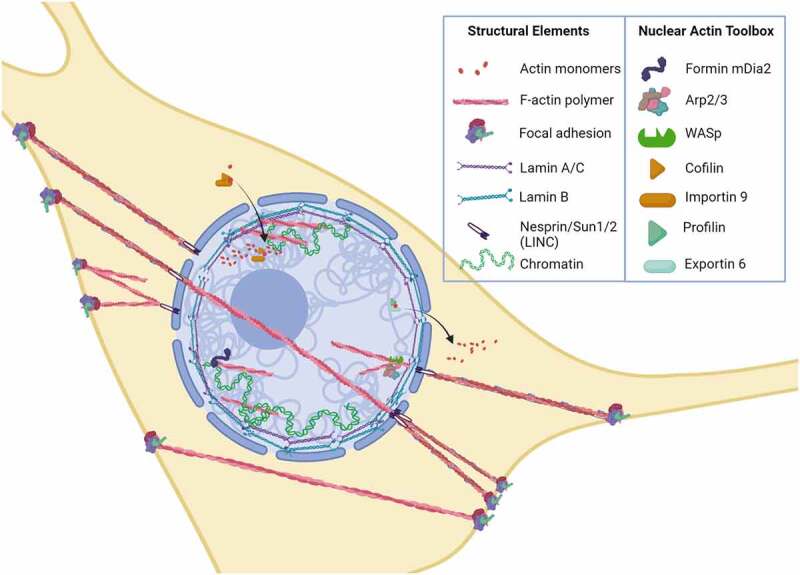
Actin tool box defines actin structure into the nucleus.

The nucleus represents the largest and most dense organelle in the cell. Its intricate structure conveys discrete mechanical properties [4]. Extrinsic to the nucleus, nuclear form adapts to forces delivered through actin connections to the nucleus at the linker of nucleus and cytoskeleton (LINC) [5]. Mechanical forces sensed at integrin sites of cell/substrate activate RhoA that not only induce the formation and then maturation of focal adhesions, but also polymerization of monomeric actin into Factin. As forces impinge upon the plasma membrane, F-actin cables, intermediate filaments and microtubules are recruited, and can connect through LINC to transmit force into the nucleus [6,7]. As such, LINC complexes hardwire the nucleus to the cytoplasm and from there out to the extranuclear extracellular environment (Figure 1).

Actin monomers and polymers are found in the cytoplasm and in the nucleus. F-actin polymers are attached to the plasma membrane by focal adhesions and can connect to other focal adhesions or to nesprin in the outer membrane of LINC complex. F-actin can also traverse the nucleus in TAN lines that are connected from LINC to LINC. The LINC complex contains SUN1/2 proteins that penetrate through the inner nuclear membrane, eventually contacting lamin B in the outermost rim, and lamin A within. Co-transporters cofilin/importin 9 transfer actin into the nucleus, export utilizes profilin/exportin 6. Within the nucleus, DNA is wound with histones that associate with lamins, generally silenced – or heterochromatin, closest to the nuclear membrane. Actin is present within the nucleus in both monomeric and filamentous forms, and associates with chromatin. Intranuclear actin structure is modified by formins, Arp2/3 and WASp.

Intrinsic aspects of the nucleus such as B- and A-type lamins [8] as well as the heterochromatin densely packed with histones and DNA contribute to modulus and shape [9]. For example, as stem cells differentiate, their nuclei stiffen largely due to increased lamin A expression [10,11]. During stiffening, chromatin is also reorganized [12], resulting in changed proportions and types of genes in the silenced heterochromatin state [13]; this alters those genes templates accessible for directed synthesis of cell phenotype-related RNAs. Highlighting the possible interplay between lamin A/C and chromatin dynamics, depletion of lamin A/C in mesenchymal stem cells (MSC) impedes adipogenic differentiation and mRNA expression [14]. Blebbing of nuclei, which appears as responses to mechanical stress, laminopathies, and cancer, in part depends on altered lamin A/C to B ratios, resulting in localized concentrations of stress and leads to cell spreading [15]; as such nuclear structure can be inferred to directly contribute to cell shape. Volumetric forces generated in the nucleus during cell spreading are present even in the absence of LINC connections, actin contractility, and microtubule networks; this indicates that the nucleus is able to sense cell shape and alter its structure independent of connecting cytoarchitecture [16]. What is yet to be understood is how the highly dynamic actin structure *within* the nucleus might contribute to both its stiffness as well as interaction with lamin, and how this might affect the heterochromatin landscape.

## Mechanical force affects cytoskeletal and nuclear structure

Cells are attuned to perceive, respond, and employ mechanical signals to guide development and function. Sensitivity to mechanical signals is critical to sensing and balancing forces during the gastrulation phase of development *in vivo* [17] and continues throughout the entire span of an organism. MSCs are primary responders to these mechanical cues in vivo. MSCs differentiate to supply cells for the bone forming osteoblast as well as its terminally differentiated osteocyte, and for the marrow adipocyte that serves as an energy storage depot [18]. While MSC in culture can be directed into multiple lineages [19], in adult organisms they largely supply progenitors for bone and fat forming cells, and for chondrocytes during fracture repair during tissue repair and regeneration [20]. We note that the biological role of MSCs during skeletal development is well defined by specific lineage-tracing fluorescent markers. Hence, the contribution of these cells to osteogenesis and adipogenesis in vivo is beyond dispute. There is greater uncertainty about the biological properties of isolated human MSCs derived from adult patients, which may not directly support tissue regeneration, but rather have trophic and immunomodulatory properties [21]. This review focuses on how mechanical forces direct lineage commitment of MSCs.

**Figure 2.**
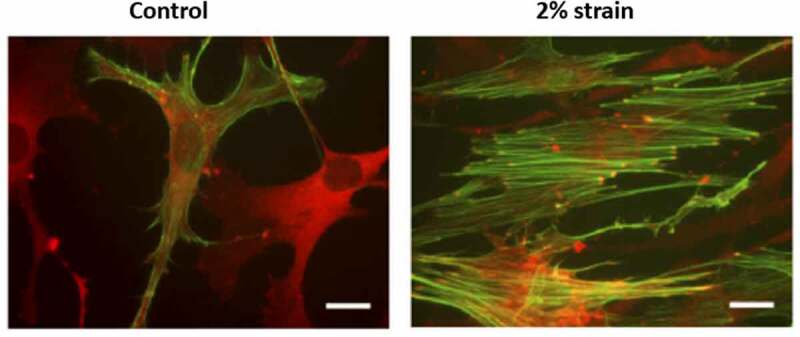
Cells respond to mechanical force by forming F-actin structures.

The formation of focal adhesions and actin polymers results in three interconnected structures collectively controlled by external forces. These molecular assemblies emerge under strain to connect to other focal adhesions [22] or from the focal adhesion to nuclear LINC contacts [6], or to travel across the nucleus as TAN lines connected by LINCs [23,24] (Figure 1). The tension produced by nuclear actin capping alters nuclear height [24], and exogenous load transmitted into the nucleus through LINC is enough to activate gene transcription [25]. Both static and dynamic forces activate RhoA through a specific G protein exchange factor (i.e., LARG). Increased RhoA activation results in further accretion of both focal adhesions and actin cabling [26,27], as demonstrated after applying mechanical strain to MSCs in Figure 2. These cytoskeletal alterations due to either mechanical loading or changes in substrate modulus can have measurable effects on nuclear stiffness of MSC and other cell types [13,28–30]. Actin structure in response to mechanical forces thus affects nuclear shape and stiffness from the outside in.

Mesenchymal stem cells were here exposed to 200 cycles of 2% equibiaxial strain over 20 min. Within 3 h, strained cells develop focal adhesions from which F-actin polymers course through the cytoplasm shown by vinculin stained focal adhesions and phalloidin stained F-actin (10 µm bar shown).

Nuclear stiffness appears to be an intrinsic property. For example, we showed that when low-intensity, high-frequency mechanical forces (‘LIV’ = 0.7 g, 90 Hz signal, 20 min × 4) were applied to MSC, F-actin contractility increased, and cell modulus measured over the nucleus increased by fourfold to 5.9 kPa (*p* < 0.001) [31]. The modulus of isolated nuclei in the experimental group increased by twofold to 2.5 kPa (*p* < 0.05). While this suggests that increased cytoplasmic F-actin remodeling is the predominant determinant of the LIV-induced cell modulus change, isolated nuclei also retain some of that information as increased modulus. A large component of nuclear stiffness is determined by lamin A/C, which scales with both substrate stiffness and actin contractility during processes of differentiation [32]. To this point, long-term culture on stiff substrates increases nuclear stiffness by promoting lamin A/C expression [33]. Moreover, a recent study reported that nuclei of breast cancer cells generate vertical protrusions under apical stress fibers; when lamin A/C was overexpressed, presumably increasing nuclear modulus, apical protrusions decreased [34].

There is also evidence that nuclear stiffness can be independent of changes in lamin A/C, depending rather on heterochromatic changes in histone methylation [35,36]. Increased F-actin contractility, as induced by applied force, can alter heterochromatin marks [14,37,38]. If the mechanical deformation is large enough, the nucleus will soften to avoid damage, and this is at least partially due to decreases in trimethylated histone 3 lysine 9 (H3K9me3) [39]. As such, decreased heterochromatin leads to a decrease in the intrinsic stiffness of nuclei. Interestingly, intrinsic nuclear stiffness has direct effects on cell movement. Treatment with the chromatin de-condensing agent trichostatin A (TSA) to inhibit histone deacetylases decreases heterochromatin and thus reduced nuclear stiffness; the TSA induced decrease in nuclear stiffness then promoted the ability of breast carcinoma cells to invade dense 3D matrices [40]. Thus, that intranuclear actin contributes is predicated not only through F-actin contractility confined to the nucleus, but, as will be covered below, via actin modulation of chromatin remodeling.

**Figure 3.**
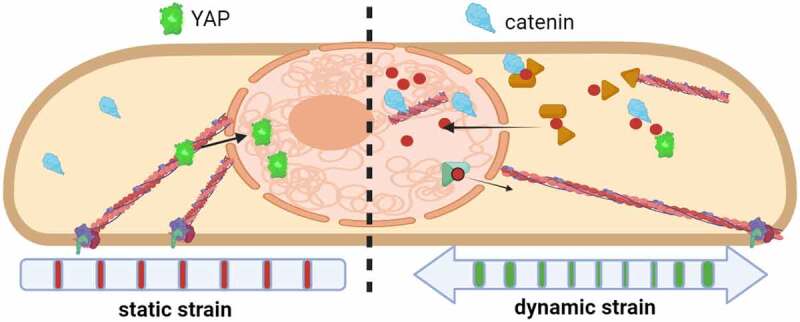
Actin transport into the nucleus is induced by dynamic mechanical force.

Indeed, application of mechanical force to isolated nuclei induces nuclear remodeling and stiffening [41] and involves multiple mutually reinforcing effects generated through chromatin condensation [39], alterations in lamin properties [32], and signaling within the nuclear envelope [42]. Along with actin-associated effects on cytoskeletal structure outside the nucleus, it is likely that intranuclear actin contributes to intrinsic nuclear stiffness. For example, despite cytochalasin D-induced depolymerization of the fibroblast actin cytoskeleton, nuclear deformation remained unchanged [43], potentially due to the influx of G-actin into the nucleus as we have demonstrated [44]. Further, we recently showed that dynamic mechanical force causes a rapid influx of actin into the nucleus, also increasing nuclear modulus by about 22% as measured by atomic force microscopy [45]. Nuclear actin transport did not occur with static strains, as represented in Figure 3. This finding suggests that inward transport of actin and its eventual disposition by members of the nuclear actin tool box together may regulate nuclear shape and perhaps regulatory events that remodel heterochromatin to control gene expression.

While application of both static and dynamic strain to MSC induces a robust actin cytoskeleton, only dynamic strain causes transport of G-actin into the nucleus [45]. Once within the nucleus, actin is subject to enzymes that presumably control polymerization. Nuclear actin influx also carries β-catenin into the nucleus. Static strain, which does not measurably affect nuclear actin level, causes YAP influx to a greater degree than does dynamic strain. In this way, nuclear actin transport serves as a mechanoresponse mechanism.

## Transport of actin into the nucleus

Actin is found within the nucleus and is critical for many nuclear processes, including gene transcription through interaction with polymerases [46], repair of DNA damage, and regulation of chromatin remodeling complexes [47]. Several excellent reviews detail aspects of nuclear actin transport, function, and direct regulation of transcription [48–50]. The model that has emerged is that actin monomers and dimers access the nucleus through an energy-dependent co-transfer involving cofilin and importin 9 [51].

Many details of the mechanism by which actin is relocated to the nucleus have come into greater focus. Actin does not have an identifiable nuclear localization signal, but its association with cofilin depolymerizes F-actin at slow-growing ends to create new barbed ends. Because cofilin has a nuclear localization signal [52] and interacts with the small GTPase Ran [49], the increased generation of actin monomers bound to cofilin permits energy-dependent nuclear transfer of actin. The actin-cofilin complex requires association with importin 9 to transport the cargo through the nuclear pore complex, and loss of either co-transporter prevents nuclear actin transport [44]. Once inside the nucleus, cofilins associate with actively transcribing genes where delivery of actin cargo supports RNA polymerase activity [53]. Actin export from the nucleus requires the co-transporters profilin and exportin 6, thus completing the actin supply chain between cytoplasm and nucleus. Profilin may also inhibit actin assembly within the nucleus by enhancing the binding of actin to exportin 6 [54]. The model that actin access to the nuclear space is controlled by disassembly of F-actin in the cytoplasm is further supported by our finding that dynamic strain induction of actin remodeling increases the supply of actin monomers for transfer [45]. Consequently, mechanical force not only controls the compartmentalization of G-actin into F-actin, but affects nuclear actin availability through regulation of actin’s nuclear import.

## A nuclear actin tool box supports actin polymerization and branching within the nucleus

Because the nucleus is very dense, visualization of actin structure inside remains a challenge, and typical phalloidin staining does not reveal many recognizable actin cables [55]. Despite this limitation, a preponderance of evidence indicates that actin remodeling occurs within the nucleus. This inference has stimulated the quest for understanding the physiological relevance of intranuclear actin organization. Importantly, virtually all of the generally accepted members of the actin tool box that allow polymerization, depolymerization, and branching of actin monomers are found within the nucleus [56]. This nuclear actin tool box includes cofilin (encoded by CFL1 and CFL2), diaphanous formins (encoded by the mDia1/DIAPH1 and mDia2/DIAPH3 genes), several actin-related proteins (e.g., Arp2, Arp3, and Arp4), and Wiskott–Aldrich syndrome family proteins (e.g., WASp, Wash, WASHC1), along with co-factors regulating import (cofilin, importin 9) and export (profilin, exportin 6) (Figure 1).

Formins (mDia1 and miDia2) expedite primary actin filament assembly by catalyzing end-on-end actin polymerization [57], which is also regulated by nuclear substrate availability. In bone marrow-derived MSCs, we found that mDia2 predominates within the nucleus while mDia1 is largely restricted to the cytoplasm [58]. The Arp2/3 complex located at membrane surfaces controls the emergence of secondary actin fibers angling out from the primary actin filament [59], functioning similarly in the nucleus [60,61]. Arp2 and Arp3 are activated by WASp and Wash, which when near the cell membranes support establishment of protrusions and lamellipodia for cell migration [62]. This set of actin-modifying proteins is found within the nucleus, where nuclear Wash has been observed to interact with lamin B and constitutive heterochromatin [63], presumably located near the inner nuclear membrane to promulgate secondary branching [64]. Interestingly, Arp4 has been shown to inhibit Factin formation in nuclei [65]. These data collectively establish the existence of a nuclear actin tool box and open the question of what purpose structural actin has within the nucleus.

Several reports indicate that the polymerized state of intranuclear actin guides targeting of some transcription factors. For instance, MLK1 (i.e., MAL) binds monomeric actin, thereby preventing its binding to and coactivation of serum response factor [66]: once MLK1 has trafficked into the nucleus, formin-activated actin polymerization ensures MLK1 retention where it promotes serum induced transcriptional responses. A role for nuclear F-actin complexes in protection of DNA has recently been discovered by Lamm et al. as actin polymers promote fork repair during the stress of cell replication [60]. Using super-resolution imaging, the group showed that WASp and Arp2/3 converged to produce Factin structures that expanded nuclear volume, causing DNA foci experiencing stress replication to mobilize to the nuclear periphery where repair could occur. Hence, actin assembly appears to play a localized role that counteracts mechanical stress, while stabilizing molecular machineries for RNA synthesis or DNA repair.

Actin rods can be observed in the nuclei of a small subset of cells in response to heat shock, hypoxia, or cell toxins, depending on the degree of stress [67]. These rods do not organize into a more elaborate interconnected actin structure that is visually obvious. However, it is plausible that a broader actin network may indeed occur. For instance, the inner ring of the nuclear pore is linked to filaments within the nucleus; while the composition of these networked filaments is unclear, they collapse when actin is depolymerized by latrunculin A and form a more open structure when the *Xenopus* oocyte nuclei is treated with Jasplakinolide to stabilize actin filaments [68]. These findings are consistent with nuclear actin having a role in intranuclear trafficking of molecules entering through the pores, perhaps by providing a chromatin-free area. Treatment with latrunculin also was shown to increase nuclear binding of an antibody that recognizes G-actin surface, but whose epitope is buried in the F-actin polymer [69]. In addition, probes that locate filamentous actin move slowly, suggesting that they are part of a larger viscoelastic structure [70].

Visualization of F-actin fibers in the nucleus is a challenge for the field. Phalloidin-stained actin filaments (a gold standard for localizing actin polymers) are rarely seen in normal nuclei or only in small percentages of stressed nuclei. This may be because the polymers are too short to be recognized [55]. Further, phalloidin’s affinity for cofilin may decrease its association with actin within the small nuclear space, and it is possible that full permeabilization of the nucleus required for phalloidin penetration might interfere with actin structural elements [71]. Using labeled actin has shown instances of both rod and filamentous particles, but only in a few cells [44]. Other F-actin markers involve tagging actin binding domain proteins with fluorescent markers. LifeAct-GFP, perhaps the most widely available actin visualization marker, unfortunately has a propensity for high background fluorescence and also binds globular G-actin as has been noted in [71]. A similar type of probe, an anti-actin-chromobody-GFP-NLS, used to monitor assembly of nuclear F-actin structures during mitotic exit is best visualized by superresolution microscopy [72]. Visualization still remained limited, even though these authors showed that impairing nuclear actin assembly interfered with the nuclear volume expansion in early mouse embryos. This result provides evidence for the physiological importance of actin dynamics in the nucleus. The landmark report establishing a role for dynamic nuclear F-actin, however poorly quantitated, is the 2013 report of Baarlink et al. [73]. There, formin-induced polymerization of actin within the nucleus was critical to subsequent transcriptional effects of serum response factor. Further, the authors were able to show nuclear filaments in live NIH3T3 cells. Broader replication of such technically demanding observations, including delivery and quantification, within the dense nucleus has not yet been fully achieved with current methodologies.

## Mechanical force guides mesenchymal stem cell differentiation through remodeling of actin structure

The ability to sense the environment and transmit force into the nucleus affects phenotypic endpoints of MSCs. MSCs, and certainly stem cells of other embryonic derivations, exist in many adult tissues and likely subscribe to general rules for attaining differentiated states. Our laboratories have concentrated on the output of bone marrow MSCs. In the laboratory cultures of bone marrow, MSCs can be guided to become osteoblasts, chondrocytes, adipocytes, fibroblasts, and even myocytes when grown in appropriate media and physical conditions. In developing vertebrates, however, bone marrow-derived MSCs are more limited, preferentially adopting osteoblastic or adipogenic cell fates. Dynamic loading – or exercise – of the live skeleton promotes osteoblastogenesis and formation of new bone to withstand and adapt to loading conditions. Removal of loading, for instance, during long stays in a hospital bed or lack of loading due to nerve damage or other types of restricted motion, all lead to loss of bone and increased production of bone marrow adipocytes from the MSC pool [74]. This understanding has led to an intense study of how MSC might sense loading conditions and respond. While certainly humoral control by other cells participates in directing MSC output [74,75], there is a great deal of evidence to indicate that MSC themselves sense and respond to loading conditions.

Cytoskeletal sensing of substrate force directs MSC differentiation: plating cells on hard substrates promotes osteoblast cell fate, and soft surfaces encourage the adipocyte phenotype [19]. It has since been accepted that genetic elements within the nucleus respond to mechanical challenges indirectly through their transduction into intermediary biochemical cascades, for instance, with activation of signals such as β-catenin [76], YAP [77], and actin [45] translocation to the nucleus (Figure 3). Mounting evidence suggests that applied forces might also directly alter chromosomal conformations, thus influencing the accessibility of genetic information for binding of transcriptional enhancers or repressors [78,79]. The ultimate target of LINC connectivity and transfer of structural information is thought to be the nuclear lamin nucleoskeleton that is packed against the inner nuclear membrane. In this way, alteration of LINC by changes in intracellular forces is expected to modulate gene expression. For example, depleting LINC element Nesprin-2 disrupts the localization and reduces levels of the heterochromatin protein HP1β/CBX1 [80], which regulates H3K9me3 levels [81]. Heterochromatin loss mediated by decreased HP1 [82] levels are implicated in aging [83,84] and in premature aging syndromes [85]. In yeast, deletion of the Sun analog Csm4 unravels chromatin organization, increasing its diffusivity and preventing DNA repair [86]. Decreased HP1β levels in MSCs transduced with nonfunctional LINC complexes suggest that a disorganized nucleus experiences deregulated transcription. Interestingly, we have reported that applying daily mechanical challenge in the form of low-intensity vibration protects cells’ ability to differentiate into osteogenic and adipogenic lineages during replicative aging [87], suggesting long-term retention of LINC-mediated mechanical information inside the nucleus.

Inhibiting the cellular capacity to form a cytoplasmic F-actin structure during in vitro mechanical loading promotes adipogenesis and prevents osteogenesis [22], and also prevents load-induced generation of the β-catenin signal as necessary induction requires focal adhesion/actin networks [27]. Many other signals are induced through integrin-initiated force through plasma membrane focal adhesions that guide proliferation and differentiation [88]. In the case of β-catenin, the predominant effect in MSC may be to induce proliferation and retain multipotentiality [89], implying that actin is also involved in these processes.

The load-induced assembled F-actin network in the cytoplasm transmits substrate force to the nucleus via LINC connectivity [6,90]. Transmission of load has many resulting effects on nuclear geometry, changing height, area, and anisotropy. In an effort to link the load transmitted through actin structure to changes in MSC phenotype, we applied the depolymerizing agent, cytochalasin D. Unexpectedly, we observed that loss of actin structure did not result in transition of MSCs to an adipocyte phenotype, but rather cells became osteoblasts [44]. Our study revealed that the osteoblast transition in the presence of cytochalasin D was entirely dependent on mass transport of actin into the nucleus. Importantly, cytochalasin D is not transported into the nucleus and therefore cannot directly affect nuclear actin structure. In turn, the Arp2/3 inhibitor CK666 does enter the nucleus where presumably it inhibits secondary actin polymerization. When CK666 was added to MSC cultures in the presence – or absence – of cytochalasin D, the Arp2/3 inhibition not only prevented osteogenesis, but induced a robust adipogenic response [64]. As such, actin levels within the nucleus are critical to stem cell fate, and the extent of actin branching regulates both osteogenic and adipogenic cells fates.

Another clue to the importance of intranuclear actin structure for cell fate decisions was through effects of knockdown of mDia2, which we demonstrated to be the major diaphanous formin in the nucleus in mouse MSC, and not located in the cytoplasm [58]. Knocking down mDia2 did not alter visual cytoplasmic actin structure, where the major formin was found to be mDia1. However, knockdown of mDia2 caused a reduction in F-actin in the inner nuclear envelope as well as a decrease in nuclear modulus. These reductions were accompanied by a decrease in lamin B, but not lamin A/C. As such, inhibition of nuclear formin with changes in at least peripheral nuclear actin structure altered expression of another structural protein. Notably, decreased lamin B1 expression is thought to be a senescence effector, causing epigenetic alterations in chromatin accessibility [91]. Furthermore, this combination of effects due to mDia2 knockdown resulted in MSC entering terminal osteoblastic lineage, suggesting increased accessibility of osteoblast genes to transcriptional activators.

Interestingly, the actin polymerization state can, in principle, be controlled by an architectural regulatory function of β-catenin/CTNNB1 via interactions with α-catenin/CTNNA1 in cadherin junctions at points of cell-to-cell contact [92]. α-catenin does not bind β-catenin and actin at the same time [93]. Thus, when β-catenin is ‘activated’, it may move toward the nucleus, while also releasing α-catenin from sequestration and permitting α-catenin dimerization. In its dimeric state, α-catenin can suppress Arp2/3-mediated actin branching. As such, α-catenin’s bundling of linear actin upon activation of βcatenin may indirectly contribute to cytoplasmic actin structure [94]. β-catenin, when activated in MSC by mechanical force [95], moves to the nucleus, where it supports preservation of the stem cell state [89]. In this instance, actin is critical to force sensation at the plasma membrane, but also critical to the actual transport of actin into the nucleus [45] as noted above. While β-catenin is known to interact with the LEF/TCF transcription factors, it could also have a structural role related to actin as it has been shown to be associated with LINC and lamin within the nucleus [90].

Access of proteins necessary to nuclear functions, including regulation of gene expression, requires transport through nuclear pores whose openings are subject to physical constraints provided by the cytoskeleton. For instance, the cytoplasmic actin cytoskeleton controls nuclear localization of gene trans-acting factors as evidenced study of the Yorkie-homologues YAP and TAZ. These two proteins are mechanosensitive co-regulatory factors that bind to sequence-specific transcription factors via tryptophan (W)-containing protein/protein interaction domains (i.e., WW domain). In response to extracellular mechanical signals generated by substrate stiffness, both YAP and TAZ translocate to the nucleus [96]. YAP is excluded from polymerized actin, thus appearing in the nucleus when a robust cytoplasmic actin structure is formed in response to substrate modulus [77]. Transfer of YAP into the nucleus is consequent to the increased availability of capping factors cofilin and gelsolin which normally limit the size of actin fibers. Although it is conceivable that cofilin chaperones YAP into the nucleus, our recent experimental data indicates that YAP transfer is unaffected when cofilin is depleted [45]. Rather, it appears that the external actin cytoskeleton pulls open nuclear pores such that YAP, which lacks a nuclear localization signal [97], gains access primarily through a force mediated mechanism [98,99]. In this way, plasma membrane connections to the external environment also alter the shape of nuclear pores to regulate nuclear access.

Moreover, polymeric actin prominently influences differentiation of MSC. Transcription factor RUNX2 mediates osteogenic lineage commitment and progression along the osteoblast lineage by interacting with a cistrome of bone-related genes during differentiation [100]. A proline/tyrosine (PY) motif in the C-terminus of RUNX2 recruits YAP as a cofactor to RUNX2 binding sites to repress transcription [101]. Our data suggest that RUNX2 activation may be regulated by reducing the nuclear availability of YAP, consistent with previous studies [44]. Another possibility to explain RUNX2 (or other gene activation) is that internal nuclear structure itself controls heterochromatin, a mechanism supported by the binding of lamin A/C to DNA to silencing genes through recruitment of polycomb complexes [63,102].

## Structural potential for actin in the epigenetic landscape

Much attention is now being addressed to the physical structure of genomes with appreciation that spatial arrangements affect gene availability [103]. For example, inhibition of nuclear F-actin assembly impairs the nuclear expansion and chromatin decondensation occurring after mitosis [72]. Preventing secondary actin structure within the nucleus of MSC promotes adipogenesis [64], and inhibition of actin filament acceleration within the nucleus inhibits the expression of lamin B [58]. Because application of strain, flow, or pressure to the cell promotes cytoplasmic actin remodeling [22,95], it is likely that actin tool box members present within the nucleus are also subject to regulation by mechanical forces.

Heterochromatin, the dense and compacted nucleosomal organization of silenced, unexpressed and inaccessible genes, is generally relegated to the periphery of the nucleus. In MSCs that are in a resting or stem-like state, quiescent genes are positioned as such at the periphery of the nucleus [29]. During differentiation, genes that orchestrate lineage [104] or preserve stemness [105] exchange positions between the periphery and the nucleoplasm. Chromosomes thus move within the nucleus, and actin appears to play a significant role in this movement. Inhibition of actin polymerization decreases subtelomeric movement, resulting in genome stability [86]. For DNA repair, at the very least, actin polymerization is critical to promote chromosome mobility [106,107], along with the Arp2/3 branching complex, which is recruited to damaged chromatin [108]. Wang and colleagues recently showed that nuclear actin polymers and actin-binding proteins participate with myosin motors to move whole gene loci within the nucleus of yeast [109]. This points to gene accessibility – or perhaps to gene inaccessibility tied up in the peripheral heterochromatin – as subject to actin remodeling.

Gene silencing in heterochromatin may arise through increased contact of chromosomes to the lamins that form structures at the inner nuclear membrane allowing gene partitioning [110]. Many genes are associated with lamin through lamin-associated domains [20,111] and changes in lamin A/C during differentiation affect gene location [112]. At the very least, actin has the potential to modulate lamin gene interactions, as shown by the significantly decreased lamin B consequent to inhibition of nuclear formin in MSC [58]. Furthermore, in the lamin A mutation defining the Hutchinson–Gilford progeria syndrome, the inability of the mutated lamin to bind actin contributes to the irregular nuclear shape and dysfunctional gene expression [113]. As nuclear formin activity is required to initiate DNA replication [114], effects of this actin tool box member are projected to be multiplex.

Further, alterations in intranuclear actin level and structure may be critical regulators of gene accessibility. Recent work has shown that knockdown of β-actin, resulting in an absence of nuclear actin, causes a general decrease in gene activation through associated chromatin remodeling [115]. Moreover, the availability – or unavailability – of G-actin is well known to participate in chromatin remodeling: actin is present in the INO80 chromatin remodeling complex interestingly associated with Arp4 [55], which has recently been shown to suppress nuclear F-actin [65]. Our group showed that inhibiting Arp2/3 induces a nearly total progression of MSC into the adipogenic lineage [64], suggesting that branched actin polymers block access to adipogenic gene enhancers. Along with work suggesting that short-actin polymers are excluded from chromatin-based processes [70], it appears likely that control of actin state modifies the heterochromatin state.

Thus, beyond well-accepted roles of actin to associate and promote RNA polymerase [116], it has become apparent that actin has the capacity to directly alter the epigenetic landscape. Monomeric actin interacts with proteins in the histone deacetylase 1 (HDAC1) complex: increased concentrations of monomeric actin limits HDAC function, while loss of the monomeric pool to polymeric actin filaments allowed for a greater HDAC activity [117]. Percipalle and coworkers have contributed significantly to this field; in 2018, they published that global chromatin organization necessary for the murine cell phenotype was dependent on β-actin [118]. Recently, as noted above, they showed that absence of actin from the nucleus leads to changes in chromatin remodelers including EZH2, the active enzyme of polycomb repressive complex 2 (PCR2) [115], which is known to control MSC cell fate [119] and skeletal development [120]. We previously showed that actin transport into the nucleus caused decreased EZH2 activity and expression [64,121]. As such, changing actin levels in the nucleus leads to chromatin remodeling. EZH2 is also affected by force transmitted through actin cables outside of the nucleus [78] as well as force-activated β-catenin [89], which does complicate analysis of the ‘where’ from which Factin contractility emanates. However, as dynamic force also promotes the nuclear import of actin, accruing research suggests that nuclear actin availability and structure are key components of the mechanism by which force regulates the epigenetic landscape [45].

In sum, while it is still difficult to characterize changes in actin structure after mechanical or pharmacological treatments, a wealth of data support that intranuclear actin structure is involved with chromatin remodeling – and maybe even that histone modifiers regulate actin structure. A recent study of differentiating T-cells indicated that EZH2ʹs methyltransferase activity initiates assembly of intranuclear actin polymers was shown using superresolution microscopy and computational modeling [122]. The authors concluded that EZH2 co-localized with actin filaments and components of the Arp2/3 machinery (Vav1 and Wasp). This work promises that further interactions of histone modifiers with the actin toolbox and their effects on gene access will soon be uncovered.

## Conclusions

A dynamic actin structure within the nucleus can be convincingly inferred from the functional presence of a nuclear actin tool box. The ability to transmit forces generated at the plasma membrane via F-actin cables linked into the nucleus, and over the nucleus, affects intrinsic nuclear properties. Further, in response to force, actin transport into the nucleus can rise, along with the transfer of mechanoresponse molecules that activate gene expression. Actin is necessary for gene transcription, and its partition into monomeric versus polymeric forms has been shown to affect the differentiation of stem cells. Within the nucleus, the presence of structural actin, and the ability of actin to cause shape change is predicted to regulate both chromatin organization and the activity of enzymes that mediate heterochromatin formation. As such, nuclear actin dynamics represents a new mechanism whereby architectural elements linked to actin structure influence epigenetic regulation of gene expression.
